# Modelling and evaluation of light railway system’s noise using neural predictors

**DOI:** 10.1186/s40201-015-0173-3

**Published:** 2015-03-17

**Authors:** Selçuk Erkaya, Abdurrahman Geymen, Bülent Bostancı

**Affiliations:** Engineering Faculty, Mechatronics Engineering Department, Erciyes University, Kayseri, 38039 Turkey; Engineering Faculty, Geomatics Engineering Department, Erciyes University, Kayseri, 38039 Turkey

**Keywords:** Noise mapping, Neural networks, Radial basis function

## Abstract

**Background:**

Noise is defined as a sound or series of sounds that are considered to be invasive, irritating, objectionable and disruptive to the quality of daily life. Noise is one of the environmental pollutants, and in cities it is usually originated from road traffic, railway traffic, airports, industry etc. The tram is generally considered as environmentally friendly, namely non-polluting and silent. However complaints from residents living along the tramway lines prove that it may sometimes cause annoyance. In this study, a Global Pointing System (GPS) receiver for determining the sampling locations and a frequency based noise measurement system for collecting the noise data are used to analyse the noise level in the city centre. Both environmental (background) and tram noises are measured.

**Results:**

Three types of neural networks are used to predict the noises of the tram and environment. The results of three approaches indicate that the proposed neural network with Radial Basis Function (RBF) has superior performance to predict the noises of the tram and environment.

**Conclusions:**

For making a decision about transportation planning, this network model can help urban planners for evaluating and/or isolating the tram noise in terms of human health.

## Background

The first step of effective noise reduction is to define the main sources of the noise generation. Noise has a clear effect upon person’s health in terms of physiological and physical approaches. For example, negative effects on sense of hearing may be considered as a physical effect. These effects may cause temporary or permanent hearing loss. Physiological effects may include increase in blood pressure, cogwheel rigidity, stress, irregular heartbeat, pupil enlargement, insomnia, and tachyon etc. The application of neural networks has been rapidly expanded over the last two decades due to the progress in computer modelling and sensor technologies [1]. In case of system identifications using neural networks, the main purpose is usually to define a dynamically valid model which can be used for system analysis.

Modelling and prediction of noise by using classical approaches is a very complex and nonlinear process, due primarily to involvement of several factors on which noise level depends. To overcome these problems, researchers and acoustical engineers have applied the neural networks (NN) in the field of noise prediction. A literature review concerning the application of neural networks in traffic noise prediction was presented [2]. A back propagation neural network (BPNN) model was used to predict the noise caused by urban traffic. Five characteristics as the number of cars, trucks and motorcycles, the average height of buildings and the road width were used as input to model the equivalent sound pressure level as output [3]. Mathematical logarithmic, statistical linear regression and neural models were presented to predict the maximum A-weighed noise level (L_A,max_) for the Tehran–Karaj express train. Measurements were obtained from sampling locations at distances of 25 m, 45 m and 65 m from the centreline of the track and at a height of 1.5 m [4]. Another study of same research group presented an artificial neural network model to predict hourly A-weighted equivalent sound pressure levels for roads in Tehran at distances less than 4 m from the nearside carriageway edge. Data were obtained from 50 sampling locations near five roads [5]. An experimental study was designed to assess the effect of road traffic noise on human performance [6]. A procedure for evaluating the noise quality of a cooling system (HVAC system), which frequently works during a day, was studied by using neural network structures [7]. Both experimental and simulation analyses were implemented together. For predicting the sound absorption coefficients of a sandwich structure nonwoven absorber, a general forecasting method was performed using general regression neural network (GRNN) [8]. Artificial neural network model was proposed to estimate the weighted sound insulation index value of wooden windows based on a limited number of windows parameters [9]. BPNN and GRNN models were used to predict the construction noise in Kuwait by considering the 33 construction sites. The GRNN model was superior to the BPNN model in its accuracy of predicting construction noise [10]. A neuro-adaptive active noise control approach with radial basis function neural network (RBFNN) was introduced for both modelling and controlling the system [11]. The feasibility and accuracy of network modelling for road traffic noise prediction was tested during the uninterrupted and interrupted traffic flow conditions [12]. Sixteen locations were identified by neural model comprising the feed forward negative back propagation algorithm. A procedure with neural predictor was proposed to test and evaluate the sound quality of cars with varying running speed [13]. Also, a robust noise analyser with NN was introduced to model and evaluate the joint noise of an industrial robot [14]. A Multi-Layered Perceptron (MLP) neural network model with Levenberg-Marquardt (LM) learning algorithm was developed for urban noise. 289 data from streets in Granada, Spain were obtained. The neural network model included 25 input variables to model the sound pressure level [15]. Noise measurements were made for 16 relevant outdoor points in the central campus area of Yildiz Technical University, Istanbul, Turkey. Artificial neural networks were used to model the variation of noise levels from traffic around the campus area. Six variables in input matrix, that is, sampling location, geographical situation, wind speed and direction, air temperature and relative humidity, and time of day were used to predict the sound pressure level [16].

The goal of this study is to present a neural network based approach to predict the level of environmental and tram line noises. Three types of neural predictor, which are extensively preferred in literature, are used for exact modelling. This paper is organized in the following manner. Section 2 outlines the methods, that is, working area, measurements and neural models. Results and discussion are given in section 3. Conclusions are outlined in section 4 for readers.

## Methods

### Working area and measurements

**Figure 1:**
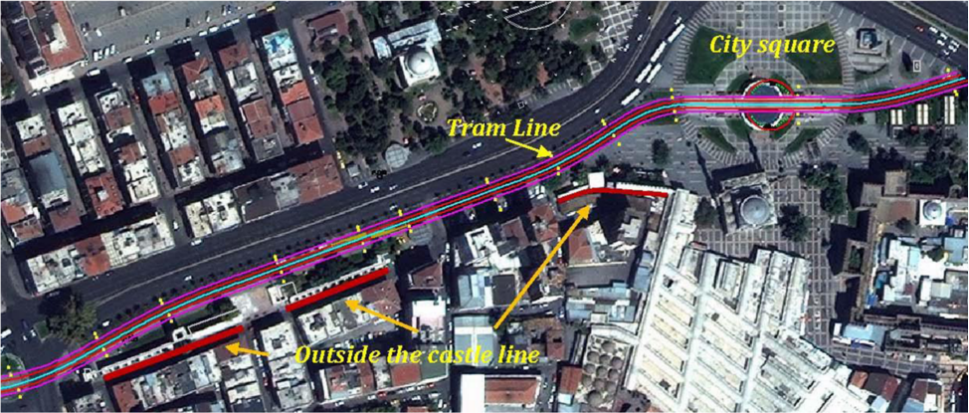
**Working area.**

Kayseri is a large and industrialized city in Central Anatolia, Turkey. It has got remarkable history and population around 1 Million. The transportation within the city relies mainly on buses, and personal vehicles. A light rail transit system called KAYSERAY was constructed and completed by the end of 2009. According to “Transportation Master Plan in Kayseri”, 82,7% of the local transportation was realized by public transportation. When the rail system was put into service in 2009, the rate of the public transportation usage was increased [17]. In order to investigate and evaluate the noise effect of the rail system on people, noise measurements near the city centre, where the traffic was highly intense in terms of the public transportation, was performed. Noise measurements were carried out between 11.1 and 11.6 kilometres of rail system in Kayseri. This area, given in Figure 1, is in the city centre and has got some historic buildings such as inner and outer towers formed by the surrounding walls as well as the old markets. Rail system route passes quite close to the outer tower.

**Figure 2:**
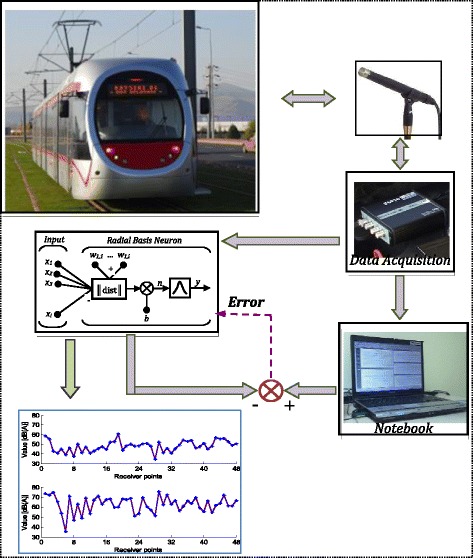
**Block diagram of experimental and simulation approaches.**

In this study, noise measurements were performed by Bruel-Kjaer (B&K) portable Pulse 3560-L data acquisition, two acoustic sensors with preamplifier and a PC [18]. Also, at the sampling locations, coordinates of locations were measured at ITRF 96 coordinate system by Astech Promark 800 GPS satellite receiver. Two noise measurements were performed at the same point as environmental and tram noises. 48 locations of tram line in the city centre of Kayseri are considered. In case of tram passing, noise measurements were performed within two days at 09:00 hours in the morning and 16:00 hours in the evening. These measurements were carried out five times. The tour interval of tram is nearly 4 minutes. The measuring area is close to tram route right. Due to the environmental conditions, such as reflection from the buildings and castle wall etc., noise values were recorded as cross-section at approximately 5 meter intervals at two points. One of them is at the right side of the tram line and the other is at the left side. Block diagram for experimental and simulation approaches is outlined in Figure 2.

### Neural network

Three types of neural network were used to model and evaluate the noise characteristics of tram and environment. Structures of neural models are given in the following subsections.

#### Radial Basis Function Neural Network (RBFNN)

**Figure 3:**
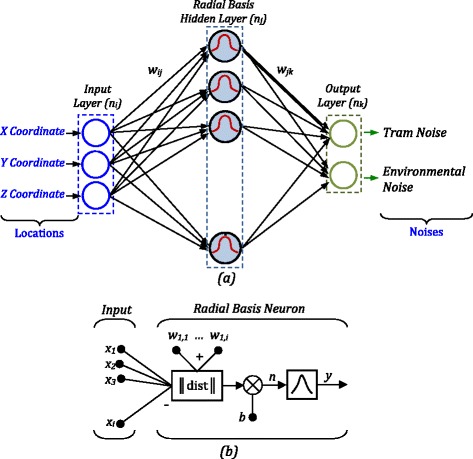
**(a) Structure of RBFNN, (b) A neuron model for** ***i*** **inputs.**

RBF neural network was firstly introduced to the thematic literature in 1988 [19]. In many engineering and science problems, this type has been widely used owing to the good convergence ability, simple structure and faster learning capability. This network type is a feed-forward network and has three layers. Input layer is the first layer and the input signals (*x*_*i*_) go from this layer to second layer. Three characteristics, that is, X, Y and Z coordinates of measured points, are considered in input matrix. In addition, tram type, tram velocity, air velocity and direction can be considered in input matrix for more stable network model. However, these characteristics have nearly zero changing. The sampling points are in the city centre and the traffic intensity is nearly similar in every hour for the daytime. Therefore, these effects were not considered in the current study. Hidden layer composed of RBF neural units is the second layer. The third layer is named as output layer, and the neurons of this layer have got linear transfer functions. Output matrix includes the tram and environmental noises. The neural network toolbox of MATLAB was used for developing the proposed network models [20]. Figure 3 outlines the RBFNN model. Hidden layer has got a non-linear Gaussian function,

1 $$ {a}_j= \exp \left(-\frac{v_j^2}{2{\sigma}_j^2}\right) $$

where *a*_*j*_ is defined as output of the *j*^*th*^ node in the hidden layer. *σ*_*j*_ is a width of the neuron *j*, *v*_*j*_ is given as Euclidean norm of the distance between input vector and neuron centre calculated as,

2 $$ {v}_j(x)=\left\Vert x-{c}_j\right\Vert =\sqrt{{\displaystyle \sum_{i=1}^r{\left(x-{c}_{j,i}\right)}^2}} $$

where *c*_*j*_ is defined as a centre of the unit *j*. The width of a unit is considered as the root mean square distance to the nearest unit *j*. For the *j*^*th*^ unit, the width ${\sigma}_j$ is defined as

3 $$ {\sigma}_j={\left(\frac{1}{\varepsilon }{\displaystyle \sum_{h=1}^{\varepsilon }{\left\Vert {c}_j-{c}_h\right\Vert}^2}\right)}^{1/2} $$

where *c*_*1*_*, c*_*2*_*, . . . , c*_*ε*_ outline the nearest unit centres to the unit *j*. The output value is given as

4 $$ {y}_k={\displaystyle \sum_{j=1}^s{d}_{jk}{a}_j} $$

where *y*_*k*_ defines the *k*^*th*^ subsection of the *y* in the output layer, *d*_*jk*_ is the weight between neuron of *j*^*th*^ hidden layer and neuron of *k*^*th*^ output layer. The number of hidden units is a crucial factor for determining the predictive properties of the network structure and is automatically calculated until the desired value of error is reached. In order to evaluate the best predictive property, various numbers of RBF units are used in network model. The number and width of RBF units are determined by hidden layer. After processing in hidden layer, the network model has got one weight value connected to the output layer. A linear squares regression algorithm is used to train the weight of output layer [21]. Mean square of the error (MSE) is considered as a performance measuring index of neural model.

#### Multi-Layered Perceptron Neural Network (MLPNN)

A MLPNN structure is used as another network model to predict and evaluate the tram and environmental noise characteristics. Each layer has got linear or nonlinear neurons and each individual neuron sums its weighted inputs and gives an output by means of a nonlinear activation function with a bias. In the current study, tangent sigmoid activation function is preferred in the nonlinear neurons,

5 $$ f(x)=\frac{1}{1+{e}^{-x}} $$

A normalized between 0 and 1 is carried out for the values of training and testing data. In order to minimize the convergence errors between desired and actual outputs of the neuron, neural model weights are updated by Levenberg-Marquardt learning algorithm. The update rule for the network weights according to Gauss–Newton (GN) method, which is a bridge between GN method and gradient descent algorithm, is outlined as:

6 $$ \Delta \omega =-{\left[{\nabla}^2E\left(\omega \right)\right]}^{-1}\nabla E\left(\omega \right) $$

where ∇^2^*E*(*ω*) is the Laplacian of the energy function and also referred to as the Hessian matrix. The Hessian term can be given,

$$ {\nabla}^2E\left(\omega \right)={J}^T\left(\omega \right)\kern0.24em J\left(\omega \right)+S\left(\omega \right) $$

where;

7 $$ S\left(\omega \right)={\displaystyle \sum_{i=1}^N{e}_i\left(\omega \right)\kern0.24em {\nabla}^2{e}_i\left(\omega \right)} $$

where *e*_*i*_*(ω)* is the error vector of the neural network for pattern *i* and *J(ω)* is the Jacobian matrix. By using the Taylor expansion, *S(ω)* is nearly considered as zero for the GN method. *S(ω)* comprises the second derivatives of the network error relative to the network weights. Since the number of computations increases exponentially with the size of the network, this term is very expensive for computing. The combination of the above equations gives the update rule of the GN method as,

8 $$ \Delta \omega =-{\left[{J}^T\left(\omega \right)\kern0.36em J\left(\omega \right)\right]}^{-1}\;{J}^T\left(\omega \right)\kern0.36em e\left(\omega \right) $$

By considering the above introduction, the LM modification to the GN method is outlined as,

9 $$ \Delta \omega =-{\left[{J}^T\left(\omega \right)\kern0.36em J\left(\omega \right)+\lambda I\right]}^{-1}{J}^T\left(\omega \right)e\left(\omega \right) $$

If the *λ* is large, Eq. (9) approximates gradient descent. Otherwise, for a small *λ*, the algorithm approximates the GN method. If the *λ* is adaptively adjusted, the LM algorithm can make provision between its two extremes, that is, the gradient descent and the GN algorithm. Therefore, the advantages of gradient descent and the GN algorithms can be combined by LM method.

#### Generalized Regression Neural Network (GRNN)

A GRNN consists of four layers: input layer, pattern layer, summation layer and output layer. The first layer is input layer and it has got input units. Pattern layer is the second layer and the outputs of this layer go to the third layer (summation layer). The last layer is the output layer. Any iterative training procedure as in back propagation method is not necessary for GRNN [20,22,23]. By considering the training data, this network type can approximate any arbitrary function between input and output vectors. GRNN is a method to estimate the Probability Density Function (PDF) of *x* and *y*. PDF can be derived from the data without any preconceptions about its form, the system is perfectly general. If *f(x,y)* represents the known continuous PDF of a vector random variable, *x*, and a scalar random variable, *y*, the conditional mean of *y* given *X* is given by

10 $$ E\left[y\left|X\right.\right]={\displaystyle {\int}_{-\infty}^{\infty }yf\left(X,y\right) dy}/{\displaystyle {\int}_{-\infty}^{\infty }f\left(X,y\right) dy} $$

If the density *f(x,y)* is unknown, it must be predicted from a sample of *x* and *y* observations. The probability estimator $\widehat{f}\left(X,Y\right)$ is based upon sample values *X*^*i*^ and *Y*^*i*^ of the random variables (*x* and *y)*;

11 $$ \widehat{f}\left(X,Y\right)=\frac{1}{(2p)^{\left(p+1\right)/2}{s}^{\left(p+1\right)}}\frac{1}{k}\times {\displaystyle \sum_{i=1}^k \exp \left[-\frac{{\left(X-{X}^i\right)}^T\left(X-{X}^i\right)}{2{s}^2}\right] \exp \left[-\frac{{\left(Y-{Y}^i\right)}^2}{2{s}^2}\right]} $$

where *k* is the number of sample observations and *p* is the x dimension*.* A physical interpretation of the probability estimate $$ \widehat{f}\left(X,Y\right) $$ is that it assigns sample probability of width *s* for each sample *X*^*i*^ and *Y*^*i*^, and the probability estimate is the sum of those sample probabilities [22]. Defining the scalar function $$ {D}_i^2, $$

12 $$ {D}_i^2={\left(X-{X}^i\right)}^T\left(X-{X}^i\right) $$

and performing the indicated integrations gives,

13 $$ \widehat{Y}(X)={\displaystyle \sum_{i=1}^n{Y}^{{}^i} exp\left(-\frac{D_i^2}{2{s}^2}\right)}/{\displaystyle \sum_{i=1}^n exp\left(-\frac{D_i^2}{2{s}^2}\right)} $$

Eq. (13) can be applied to problems comprising the numerical data.

## Results and discussion

**Figure 4:**
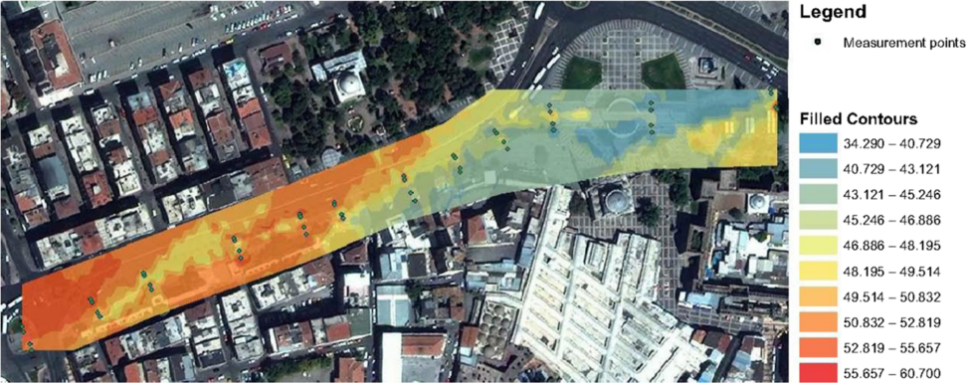
**Environmental noise map.**

**Figure 5:**
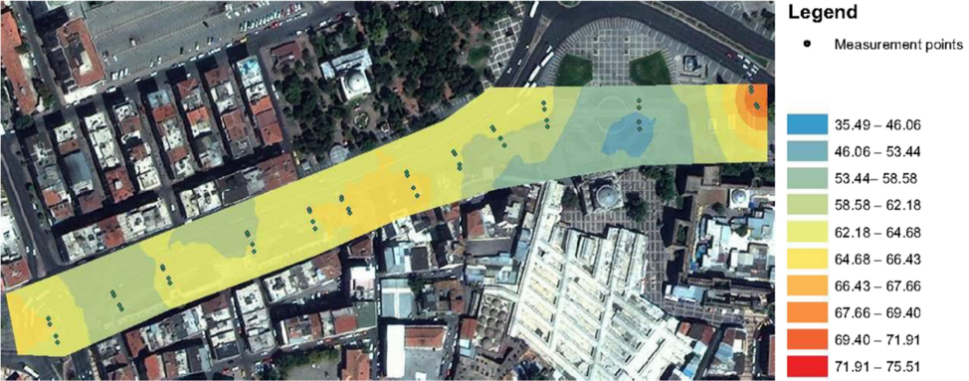
**Tram noise map.**

Neural network predictors for environmental noise, that is, background noise except for tram, and tram noise are presented in the current study. By using the interpolation technique, noise maps of environment and tram are outlined in Figures 4 and 5, respectively,

According to the directive on the assessment and management of the environmental noise accepted in 2010 in Turkey, it is stipulated that the noise level (L_day_) spread from the rail transport systems around during the day may not exceed the limit value of 65 dBA. However, by evaluating the Figures 4 and 5 together, 22 measurements at the 48 sampling points exceed this limit value.

**Figure 6:**
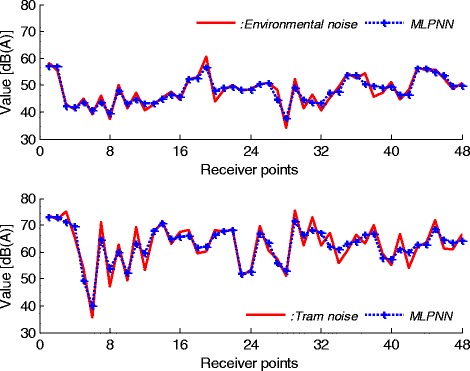
**MLPNN results for environmental and tram noises.**

After the measurements, neural network models for predicting the noise characteristics are constituted. Training and testing stages of the network structures are employed on neural network toolbox of MATLAB. 175 data are used in the training stage of the designed networks. Also, these networks are validated and tested with 52 and 48 data, respectively, in responses to input which have not been used in the training step. The percentage of these data sets is nearly consistence with literature [5,21,23]. After the training and testing stages of networks are completed, the weights are saved and used for predicting and estimating the environmental and tram noises for given an input matrix. Both prediction and experimental results are compared to each other for case of the network stability. Figure 6 gives the results of MLPNN predictor for environmental and tram noises.

**Figure 7:**
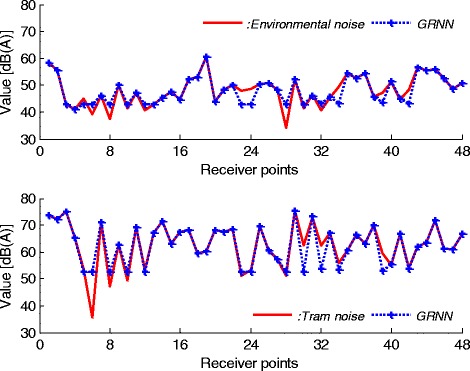
**GRNN results for environmental and tram noises.**

By evaluation of Figure 6, it can be concluded that the network outputs nearly follows the measured values. But, this MLPNN predictor is not suitable for modelling the noise characteristics. Because, there is a clear difference between NN outputs and real noise values. These differences can be observed at all points. The second predictor model is based on GRNN. The simulation results for this predictor are given in Figure 7.

**Figure 8:**
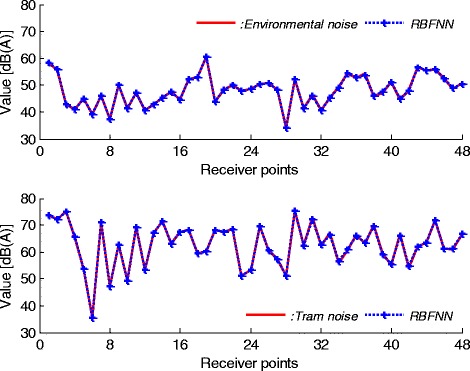
**RBFNN results for environmental and tram noises.**

As seen from Figure 7, while environmental and tram noises at some points can be predicted correctly, this evaluation for all points is not valid. There is not an exact matching between experimental and neural simulation results. This GRNN predictor is also not suitable for modelling the noise characters. The last predictor model is RBFNN. The simulation result for this predictor is given in Figure 8.

**Figure 9:**
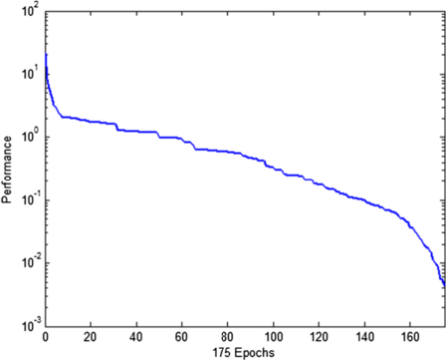
**Performance index of neural predictor varying with epoch.**

Figure 8 shows that RBFNN model has a great accuracy for predicting and evaluating the environmental and tram noise characteristics. The approximation capability of the proposed network type is very suitable for this task. This model can be used to evaluate the intermediate values of the related noises. Training history of RBFNN is outlined in Figure 9.

**Figure 10:**
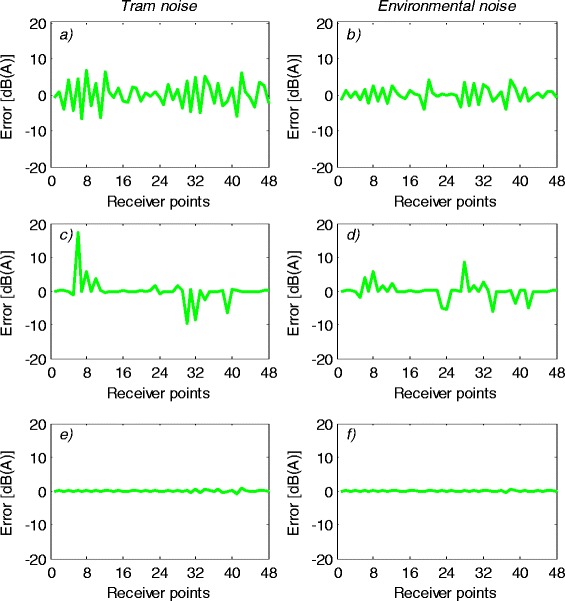
**Convergence errors of neural models, a) and b) Error for MLPNN predictor, c) and d) Error for GRNN predictor, e) and f) Error for RBFNN predictor.**

By considering the differences between measuring and modelled noise values at the same points, convergence errors of the MLPNN, GRNN, RBFNN are outlined in Figure 10. Figure 10(a) and (b) denote the convergence errors for MLPNN predictor for the tram and environmental noises, respectively. Also, these errors for GRNN predictor are outlined in Figure 10(c) and (d) for each noise, respectively. In the case of RBFNN predictor, these errors are given in Figure 10(e) and (f) for the tram and environmental noises, respectively.

As seen from Figure 10, convergence errors of the RBFNN are smaller than that of the GRNN and MLPNN predictors. This is a good reflection of modelling and predicting capabilities of the proposed RBFNN model. A good convergence was achieved by comparing the measurement results and the results from the RBFNN method. It can be concluded that the prediction of the environmental and tram noises is possible by using the RBFNN. Also, proposed neural model has a great accuracy to predict and estimate the noise characteristics under the received points. When the neglected parameters in input matrix, that is, tram type, measuring hour, tram velocity, air velocity or ground effects etc. have a considerable change, these characteristics should be considered in input matrix for more stable and accurate RBFNN predictor. These arrangements also affect the benefits and/or limits of neural model for noise prediction.

## Conclusion

In this study, a neural predictor is proposed to model and evaluate the characteristics of environment and tram noises. At the first stage of this study, the route of Kayseri light railway system, between 11.1 and 11.6 kilometres, is considered to evaluate the environmental and tram noise. 48 sampling points, where the traffic is highly intense in terms of the public transportation, are used for measuring. By considering the directive on the assessment and management of the environmental noise accepted in 2010 in Turkey, tram noises at some measured points (22 points) exceed the limit values (65 dBA).

At the second stage of this study, three types of neural networks, that is, multi-layered perceptron, generalized regression and radial basis function neural networks, are used for modelling and predicting the noise values. For training and testing of network structure, noise measurements are carried out to specify the network stability for exact modelling and predicting the noise characteristics. X, Y and Z locations for each point are taken into consideration in input matrix of network. In addition, the output matrix is constituted by environmental and tram noises. The simulation results give that the RBFNN has good convergence and high accuracy ability to predict the noise values at related points. This predictor is robust and stable to model the noise characteristics. On the contrary, other network models, that is, MLPNN and GRNN predictors are not suitable and should not be used to model and predict the noises correctly. The proposed neural predictor may be helpful to urban planners for making a decision about transportation planning. Also, these values can be used to select the correct materials for noise isolation.
